# Phage resistance bidirectionally altered antibiotic susceptibility in *Klebsiella pneumoniae* via *galE* mutation

**DOI:** 10.1016/j.ijantimicag.2026.107738

**Published:** 2026-02-07

**Authors:** Braira Wahid, Sue C. Nang, Jinxin Zhao, Zhi Ying Kho, Meiling Han, Heidi Hoi-Yin Yu, Hasini Wickremasinghe, Phillip J. Bergen, Saima Aslam, Robert T. Schooley, Jian Li

**Affiliations:** a Department of Microbiology, Monash Biomedicine Discovery Institute, Monash University, Clayton, VIC, Australia; b Division of Infectious Diseases and Global Public Health, Department of Medicine, University of California San Diego, La Jolla, CA, USA

**Keywords:** Antimicrobial resistance, Phage therapy, Phage adsorption, *galE*, Lipopolysaccharides

## Abstract

**Objective:**

Multidrug-resistant (MDR) Gram-negative bacteria, particularly *Klebsiella pneumoniae*, represent a significant threat to global health. Given the shortage of new antibiotics, bacteriophages (phages) offer a promising alternative. Understanding bacteria-phage interactions and resistance mechanisms is vital for optimising phage therapy. We aimed to investigate the resistance mechanism of an MDR clinical isolate, *K. pneumoniae* UCSD1 (designated as KpUCSD1) against a newly isolated phage ΦKpUCSD1.

**Methods:**

A phage-resistant mutant KpUCSD1R was collected at 24 h following treatment of KpUCSD1 with ΦKpUCSD1. Comparative genomic analysis identified mutations in KpUCSD1R, and complementation was conducted to evaluate the impact of these genes towards phage resistance. Spot tests and time-kill kinetics were performed to evaluate the bacterial susceptibility to ΦKpUCSD1. The role of *galE* in phage infection was evaluated with adsorption and lipopolysaccharide (LPS) assays.

**Results:**

Mutations were identified in *ubiH* and *galE* of KpUCSD1R and *galE* was crucial for phage infection, as complementing KpUCSD1R with wild-type *galE* restored its susceptibility to ΦKpUCSD1. The clean knockout strain, KpUCSD1Δ*galE*, exhibited complete loss of phage susceptibility owing to defective phage adsorption. Further analyses revealed that mutated and loss of *galE* resulted in truncated LPS. Interestingly, phage resistance imposed a fitness trade-off associated with *galE* disruption, resulting in collateral antibiotic effects, namely increased susceptibility to aminoglycosides, chloramphenicol, and polymyxins, alongside reduced susceptibility to tetracycline and meropenem.

**Conclusion:**

This study demonstrates, for the first time, that *galE* mutation in *K. pneumoniae* diminishes phage adsorption by causing truncation of the LPS. The trade-off leads to increased susceptibility to several antibiotics, offering a potential therapeutic advantage in treating MDR infections.

## 1. Introduction

Antimicrobial resistance is a significant global healthcare threat. In 2019, 4.95 million deaths worldwide were associated with antimicrobial resistance [1], and this global death toll is predicted to increase to 10 million annually by 2050 [2]. Carbapenem-resistant *Klebsiella pneumoniae* has emerged as a major global health threat and is listed in the 2024 World Health Organization global priority pathogen list [3]. *K. pneumoniae* is responsible for a wide range of nosocomial infections, including urinary tract infection, bloodstream infection, ventilator-associated pneumonia, and bacterial meningitis [4]. With increasing resistance to currently available antibiotics and a diminishing antibiotic development pipeline [5], bacteriophages (phages) are once again being considered as potential alternatives to antibiotic therapy for treating multidrug-resistant (MDR) pathogens [6].

As bacteria can deploy a variety of defence mechanisms against phage attack, the emergence of phage resistance presents a significant challenge to the success of phage therapy [7]. Various phage resistance mechanisms have been reported, such as disruption of phage binding, restriction-modification systems, and the CRISPR-Cas system [8]. The most commonly observed mechanisms involve the removal or alteration of bacterial surface components that serve as phage receptors [9]. These components include lipopolysaccharides (LPS), outer membrane proteins, cell wall teichoic acid, capsule, and flagella [10]. Notably, phage resistance associated with these bacterial structures often results in trade-off, including reduced bacterial motility, diminished biofilm formation, and increased antibiotic susceptibility [11].

Given the challenge posed by phage resistance, a comprehensive understanding of bacteria-phage dynamics is urgently needed, including the molecular mechanisms by which bacteria evade phage-mediated lysis and the physiological consequences of such resistance. In this study, we investigated the resistance mechanisms utilised by an MDR *K. pneumoniae* strain isolated from a patient against a novel lytic jumbo phage. We also examined the effect of phage resistance on antibiotic susceptibility, revealing a key phenomenon: Phage-Induced Antibiotic Susceptibility Reprogramming, characterised by bidirectional shifts in antibiotic sensitivity. Additionally, we conducted a comprehensive functional analysis to explore the role of *galE* in *K. pneumoniae*, a topic previously unexamined in the context of phage resistance. By elucidating the specific adaptive strategies employed by this high-priority pathogen to counteract phage attack, our study provides critical insights into the complex interactions between phages and their bacterial hosts, informing the development of effective phage therapies.

## 2. Methods and materials

### 2.1. Bacteria

*K. pneumoniae* UCSD1 (KpUCSD1) was examined in this study (Supplementary File 1). This MDR strain was isolated from the blood of a 70-y-old female who had undergone a combined kidney and liver transplant and had a history of recurrent urinary tract infections [12]. Phage-resistant isolates were obtained by infecting KpUCSD1 with phage ΦpKpUCSD1 at an MOI of 100. This high MOI was employed to apply strong selective pressure, ensuring that surviving cells represented true resistant mutants. Briefly, 200 μL of bacterial culture was collected, centrifuged at 18 000 × *g* for 10 min, and the supernatant was discarded. Subsequently, 50 μL of bacterial pellet resuspended in 0.9% sodium chloride was plated on nutrient agar and incubated overnight at 37°C. Hypermucoviscosity was assessed using the string test [13], and a capsule assay was performed using Maneval’s capsule staining method.

### 2.2. Phage isolation and characterisation

A lytic phage, ΦKpUCSD1, targeting KpUCSD1 was isolated from raw sewage samples following the Phage on Tap protocol [14]. The lytic activity of ΦKpUCSD1 was assessed using a direct spot test and a double-layer plaque assay, and one-step growth experiments were performed to determine its latent period and burst size using an MOI of 0.01 [15]. Phylogenetic analysis was performed using MAFFT v7.505.

### 2.3. *Genome sequencing and analysis of phage-resistant* K. pneumoniae

DNA from *K. pneumoniae* was extracted using DNeasy Blood and Tissue Kit (Qiagen) according to the manufacturer’s instructions. DNA was extracted from the phage genome using the phenol-chloroform method. Sequencing was conducted on Illumina HiSeq 2500 platform with a paired-end 150 bp library, achieving a mean coverage of ~100 × and an average read depth of 95–105 (GE-NEWIZ, Azenta Life Sciences, Suzhou, China). SPAdes v4.0.0 was used for *de novo* assembly, and subsequent annotation for bacteria and phage was performed using Prokka v1.13.7 and PHROG, respectively (Supplementary Files S2 and S3). Genetic variants in the phage-resistant mutants were investigated with Snippy for primary detection of single-nucleotide polymorphisms, whereas FreeBayes v1.3.6 and ProcaryaSV v1.1 were utilised to cross-check and verify the genetic variations. FreeBayes was employed to confirm the single-nucleotide polymorphisms and additionally check for smaller-scale variations (e.g. INDELs), and ProcaryaSV was implemented to examine larger structural variations.

### 2.4. Time-kill kinetics

Time-kill kinetics were evaluated using an initial bacterial inoculum of ~10^6^ CFU/mL. Briefly, 0.1 mL of mid-exponential-phase culture of KpUCSD1 (OD_600nm_ of 0.5, corresponding to ~10^8^ CFU/mL) was added to 9.9 mL of nutrient broth. Then, 0.1 mL of 10^10^ PFU/mL of ΦKpUCSD1 was added to achieve an MOI of 100. A 200-μL culture sample was taken immediately before the introduction of the phage (0 h) and at 2, 4, 6, and 24 h. Samples were centrifuged for 10 min at 18 000×*g* and 4°C, the supernatant was removed, and the bacterial pellet was resuspended in an equal volume of 0.9% sodium chloride. Serial dilutions were conducted in 0.9% sodium chloride, and 50 μL of appropriately diluted sample was plated on nutrient agar. Bacterial enumeration was performed following 24 h incubation at 37°C.

### 2.5. *Knockout and complementation of* galE *and* ubiH

To construct the recombinant plasmids pBBR1MCS-1*_galE_* and pBBR1MCS-1*_ubiH_*, the *galE* and *ubiH* genes were amplified from KpUCSD1 using KOD Hot Start DNA Polymerase (Novagen) with primers listed in Supplementary Table 1. The amplified products were ligated into the pBBR1MCS-1 vector using T4 DNA ligase (New England BioLabs). The ligation reaction was transformed into *E. coli* DH5 *α* via heat shock, and successful transformants were selected on LB agar supplemented with 50 mg/L chloramphenicol, followed by colony PCR. The resulting plasmids, pBBR1MCS-1*_galE_* and pBBR1MCS-1*_ubiH_*, were isolated and electroporated (1.0 kv, 5.5 ms; Bio-Rad MicroPulser) into electrocompetent KpUCSD1R. Plasmid uptake was verified by colony PCR and Sanger sequencing. KpUCSD1R transformed with the empty vector pBBR1MCS-1 served as the control strain.

The lambda red homologous recombination system was used to knockout *galE* from KpUCSD1. Splicing by overlap-extension (SOE) PCR was conducted to construct a PCR product with up-stream and downstream regions of *galE* flanking a chloramphenicol resistance cassette with flippase recognition target sites at both ends. The SOE PCR product was ligated into the suicide plasmid pGEM-T Easy-apr. The resulting plasmid was extracted using QIAprep Spin Miniprep kit (Qiagen) and transformed into KpUCSD1. Successful transformants were selected on LB agar containing 50 mg/L of chloramphenicol. An arabinose-inducible vector, pACBSR, was introduced into KpUSCD1 carrying pGEM-T Easy-apr-SOE PCR to promote homologous recombination for *galE* knockout. The *galE* knockout was confirmed via PCR and Sanger sequencing. Complementation of KpUCSDΔ*galE* was conducted by introducing pBBR1MCS-1*galE*.

### 2.6. Direct spot test, efficiency of plating, and phage adsorption

Phages (10 μL) were spotted onto a bacterial lawn (4 mL of soft agar containing 100 μL of overnight bacterial culture). The results were interpreted after overnight incubation at 37°C, based on the presence or absence of clear lysis zones. The efficiency of plating was calculated by dividing the PFU of the target strain by the PFU of the host strain. A phage adsorption assay was conducted as previously described [15]. The percentage of phage adsorbed onto the bacterial cell surface was calculated using the formula: % of free phage = *P_t_* / *P*_0_ × 100, where *P*_0_ represents the phage without bacteria and *P_t_* represents the phage with bacteria.

### 2.7. Visualisation of phage internalisation

Confocal microscopic analysis was conducted to visualise phage internalisation. The phage ΦKpUCSD1 was stained with 10 000 × SYBR Gold nucleic acid stain (Invitrogen), while KpUCSD1 and its mutants were stained with FM 4-64FX. Stained phage underwent five rounds of filtration using Amicon Ultra-15 centrifugal filter tubes to remove excess stain. The stained phages and bacteria were combined in equal volumes (50 μL each) at an MOI of 100 and incubated for 1 min at 37 °C. Subsequently, 10 μL of the mixture was placed onto a glass slide, immobilised using 2% agarose, and covered with a coverslip. The samples were imaged using a 63 × /1.40 oil immersion objective on Leica SP8 confocal microscope.

### 2.8. Antibiotic susceptibility

Minimum inhibitory concentrations (MICs) for ampicillin, ceftazidime, meropenem, ciprofloxacin, chloramphenicol, metronidazole, tetracycline, tobramycin, amikacin, and polymyxin B were determined using broth microdilution [16].

### 2.9. LPS analysis

*K. pneumoniae* isolates, KpUCSD1, KpUCSD1R, KpUCSD1Δ*galE*::pBBR1MCS-1*_galE_*, and KpUCSD1Δ*galE* were grown to an OD_600nm_ of 0.5 (~10^8^ CFU/mL) in LB broth. LPS was extracted using an LPS Extraction Kit (Boca Scientific, USA). The extracted LPS was treated with proteinase K and analysed on a 10% precast SDS-PAGE gel (Criterion TGX) for 1 h at 100 mA. Periodic acid-Schiff (PAS) silver staining was used to visualise LPS on the gel. The total polysaccharide fraction in the LPS was quantified by hydrolysing the LPS in 1% acetic acid for 1 h at 100°C [17].

## 3. Results

### 3.1. Phage ΦKpUCSD1 killing activity and emergence of resistance

**Fig. 1.**
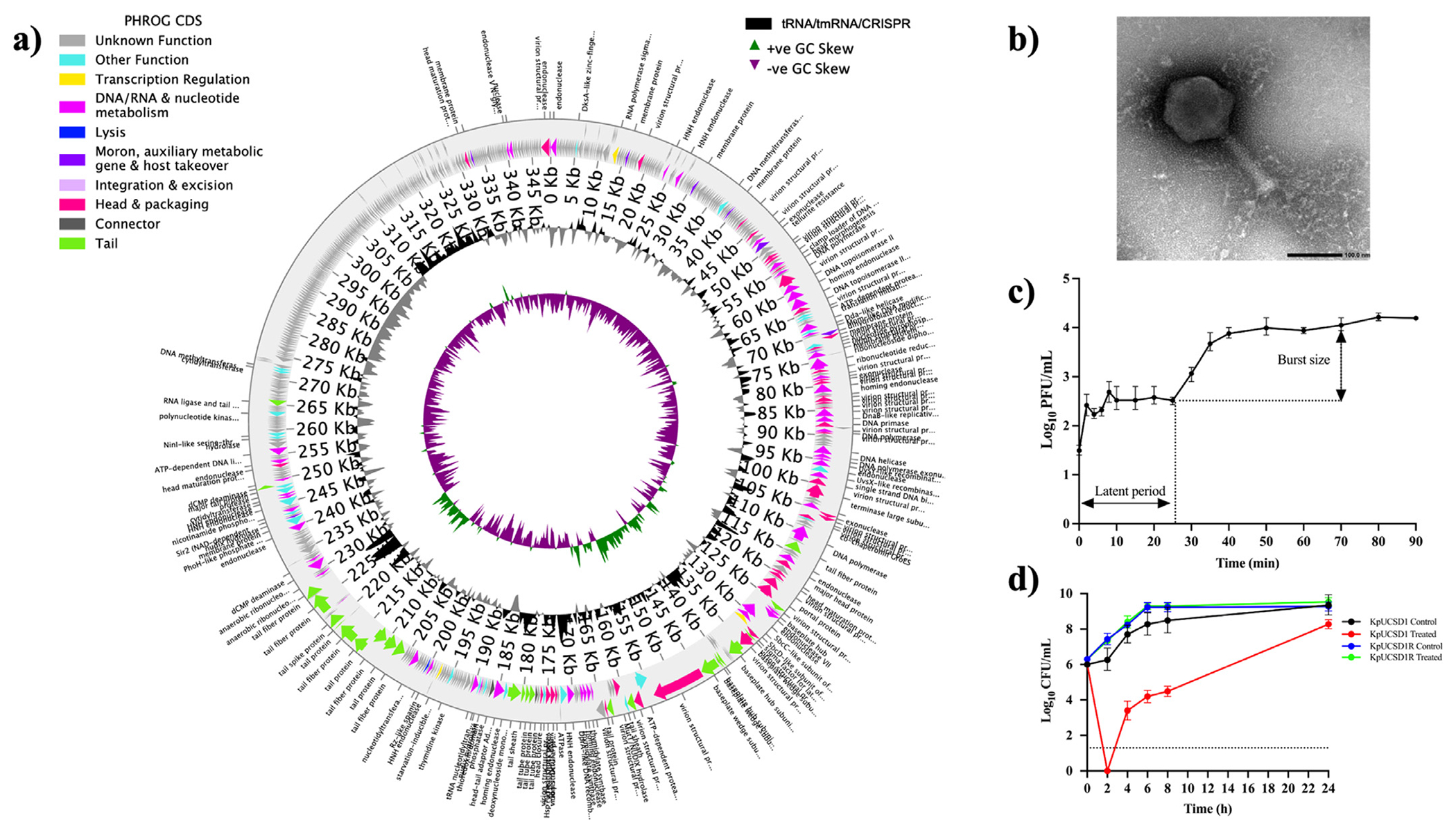
Characteristics and killing kinetics of phage ΦKpUCSD1. (A) Genomic visualisation showing predicted coding sequences (CDS). (B) TEM image of phage. The scale bar represents 100 nm. (C) One-step growth curve of ΦKpUCSD1 with KpUCSD1 as the host. (D) Growth kinetics of KpUCSD1 and KpUCSD1R in the absence (control) and presence (treated) of ΦKpUCSD1. Data are presented as mean ± standard deviation (*n* = 3). The limit of detection (LOD) was log_1 *n*_ CFU/mL = 1.30 (equivalent to 20 CFU/mL).

ΦKpUCSD1 formed clear plaques (diameter 1.5 mm) when plated on KpUCSD1 (Supplementary Fig. S1). Genomic analysis identified ΦKpUCSD1 as a jumbo-sized myophage, with a genome composed of 348 922 bp (Fig. 1A). ΦKpUCSD1 shared a high degree of sequence similarity (95%–99%) with over 60 other *Klebsiella* phages, belonging to the class *Caudoviricetes*, including several jumbo phages. TEM imaging showed that ΦKpUCSD1 had a length of approximately 238 nm, with an icosahedral capsid and a sheathed, contractile tail (Fig. 1B). A one-step growth assay revealed a latent period of 25 min and a burst size of 24.6 ± 2.81 PFU/cell (Fig. 1C). Time-kill kinetics demonstrated rapid and extensive initial killing of KpUCSD1 by ΦKpUCSD1, with no viable bacteria detected at 2 h. However, rapid regrowth was observed thereafter, reaching a level comparable to the control at 24 h (Fig. 1D). This regrowth revealed two distinct bacterial colony morphologies at 24 h: small round colonies (diameter ~0.2 cm) and large round colonies (diameter ~0.5 cm) (Supplementary Fig. S1). Unlike the original colony phenotypes, these colonies were dry, rough, non-mucoid, and produced a negative string test (Supplementary Fig. S2). The isolated small round colonies remained partially susceptible to ΦKpUCSD1, as indicated by the formation of a turbid lysis zone. No lysis zone was observed when ΦKpUCSD1 was plated on mutants with the large colony phenotype, and this derivative isolate was therefore designated KpUCSD1R. The growth of KpUCSD1R was unaffected by ΦKpUCSD1, demonstrating complete resistance. The capsule morphology of KpUCSD1R appeared similar to that of KpUCSD1 (Supplementary Fig. S3).

### 3.2. *Resistance to ΦKpUCSD1 was associated with dysfunctional* galE

**Fig. 2.**
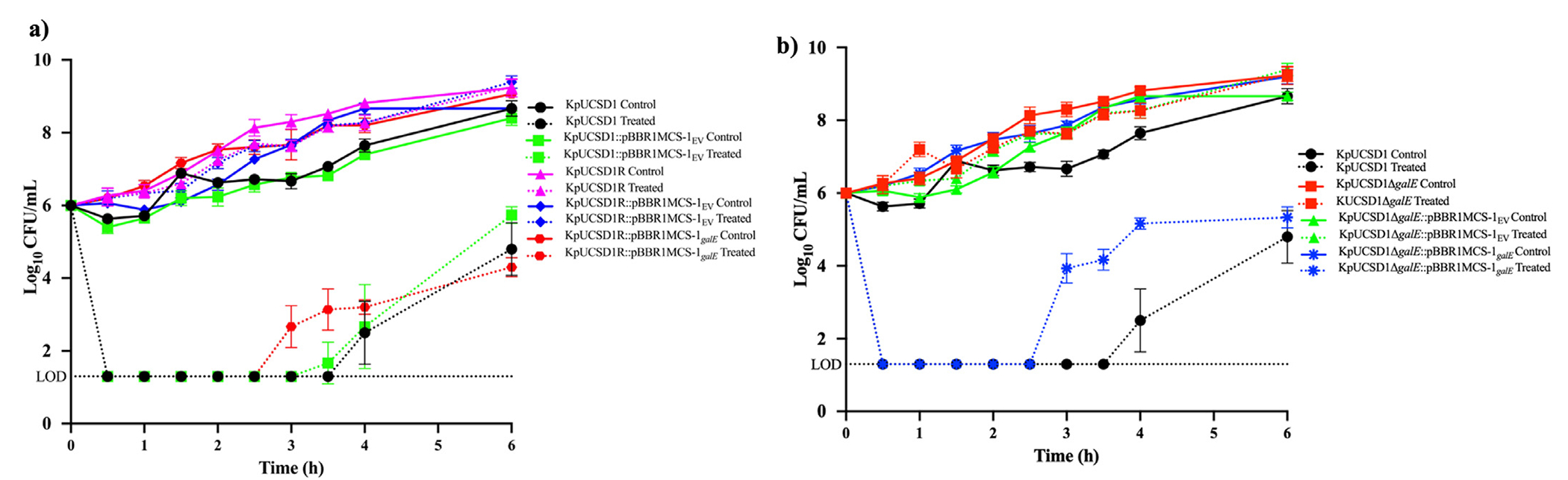
Growth kinetics of KpUCSD1 and its derivatives in the absence (control) and presence (treated) of ΦKpUCSD1. (A) Growth curves of wild-type KpUCSD1, the phage-resistant mutant KpUCSD1R, and their respective transformants carrying either the empty vector (pBBR1MCS-1_EV_) or plasmid harbouring the wild-type *galE* (pBBR1MCS-1_*galE*_). (B) Growth curves of the *galE* knockout mutant (KpUCSD1 Δ*galE*) and the respective transformants carrying either the empty vector (pBBR1MCS-1_EV_) or plasmid harbouring the wild-type *galE* (pBBR1MCS-1_*galE*_). Data are shown as mean ± standard deviation (*n* = 3). The limit of detection (LOD) is indicated by the dashed line and corresponds to log_1 *n*_ CFU/mL = 1.30 (equivalent to 20 CFU/mL).

Genome sequencing identified a frameshift mutation in *galE* (involved in LPS synthesis and membrane biogenesis) and a missense mutation in *ubiH* (involved in ubiquinone biosynthesis) in KpUCSD1R (Table 1; Supplementary Files S4 and S5). Based on the hypothesis that these genes may play a role in effective phage infection, we firstly introduced functional *galE* or *ubiH* into KpUCSD1R. A direct spot test showed clear lysis zones when ΦKpUCSD1 was plated on KpUCSD1R carrying pBBR1MCS-1*_galE_*, but no lysis zones were observed with that harbouring pBBR1MCS-1*_ubiH_*, confirming that complementing the resistant strain with a functional *galE* restored phage infection (Supplementary Fig. S4). To exclude potential secondary effects from other mutations in KpUCSD1R, we constructed a *galE* knockout mutant from the wild-type KpUCSD1, designated KpUCSD1Δ*galE*. Time-kill kinetics showed that ΦKpUCSD1 was ineffective against KpUCSD1Δ*galE*, and the complemented strain KpUCSD1Δ*galE*::pBBR1MCS-1*_galE_* regained susceptibility to ΦKpUCSD1 (Fig. 2).

### 3.3. *Dysfunctional* galE *hindered the adsorption of phage ΦKpUCSD1*

**Fig. 3.**
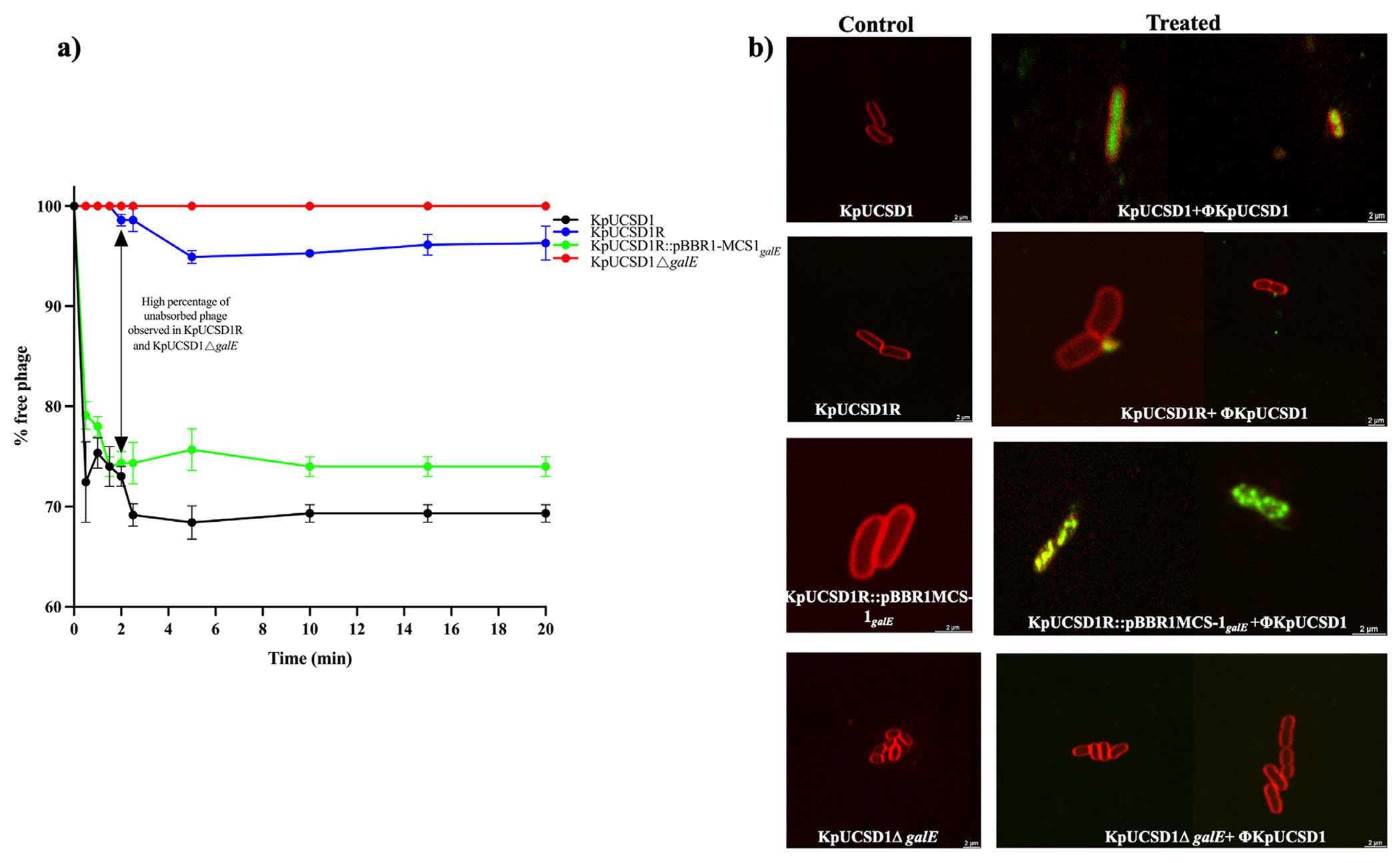
Adsorption profiles of ΦKpUCSD1 to KpUCSD1 and its derivatives. (A) Adsorption of ΦKpUCSD1 to KpUCSD1, KpUCSD1R, KpUCSD1R::pBBR1MCS-1_*galE*_, and KpUCSD1 Δ*galE*. Data are presented as mean ± standard deviation (*n* = 3). (B) Representative fluorescence microscopy images showing phage adsorption. Bacterial membranes were stained with FM 4-64FX (red), and ΦKpUCSD1 was labelled with SYBR Gold (green). A green signal represents phage particles bound to or internalised within bacteria. Scale bar = 2 μm.

Rapid adsorption of ΦKpUCSD1 was observed with KpUCSD1 as the host strain, reaching a maximum adsorption of 31.8 ± 0.99% at 2 min. In contrast, KpUCSD1R showed significantly lower adsorption, with only 5 ± 0.63% maximal adsorption observed at 5 min. Complementation of KpUCSD1R with a functional *galE* restored adsorption levels comparable to the wild-type. Notably, ΦKpUCSD1 adsorption was completely inhibited in KpUCSD1Δ*galE* (Fig. 3A). Confocal microscopy revealed hindered phage adsorption in KpUCSD1R and KpUCSD1Δ*galE*, evidenced by the absence of green fluorescence intracellularly, showing that phages could not enter these cells (Fig. 3B). In contrast, KpUCSD1 and KpUCSD1R::pBBR1MCS-1*_galE_* exhibited intracellular green signals, indicating effective phage adsorption. These results demonstrate that *galE* is essential to phage adsorption and infection.

### 3.4. *Dysfunctional galE resulted in truncated LPS in* K. pneumoniae

**Fig. 4.**
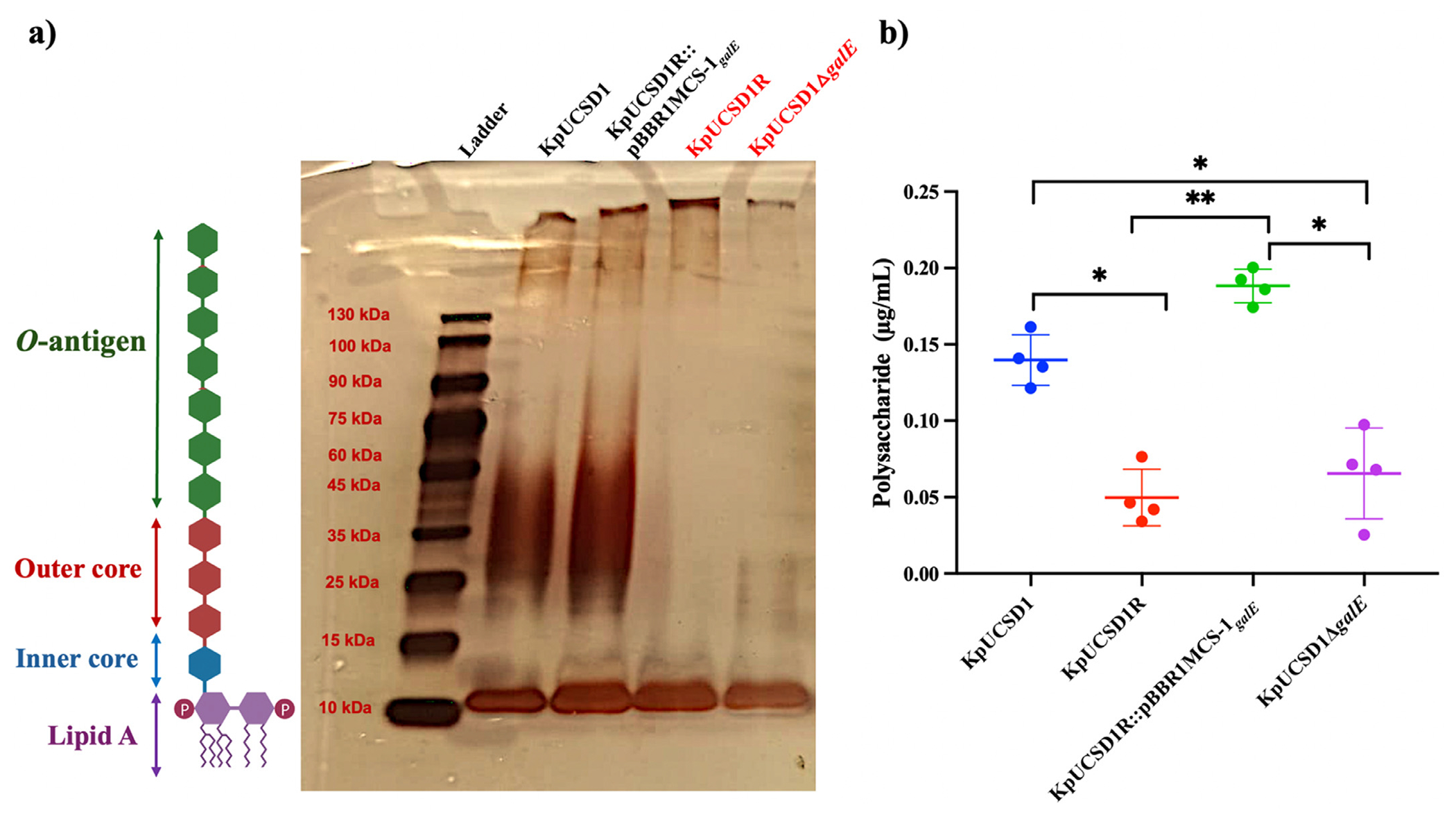
LPS profiles of KpUCSD1 and its derivatives. (A) SDS-PAGE gel image showing LPS banding patterns. KpUCSD1 and the complemented strain KpUCSD1R::pBBR1MCS-1_*galE*_ display high-molecular-weight bands corresponding to intact LPS with long *O*-antigen chains (smooth phenotype). In contrast, the phage-resistant KpUCSD1R and KpUCSD1 Δ*galE* exhibit truncated LPS lacking extended *O*-antigen repeats (rough phenotype). (B) Quantification of polysaccharide fraction of LPS. Data are presented as mean ± standard deviation (*n* = 4). Statistical significance is represented by *P < 0.05 and **P ≤ 0.01.

Lower bands (~10 to 20 kDa) in the silver staining image, representing LPS core oligosaccharide and lipid A, were present in all *K. pneumoniae* strains examined (Fig. 4A). The high molecular weight smeared pattern present in KpUCSD1 and KpUCSD1R::pBBR1MCS-1*_galE_* suggested the presence of both short and long *O*-antigen chains in these phage-susceptible strains. In contrast, LPS samples from the phage-resistant KpUCSD1R and KpUCSD1Δ*galE* demonstrated defective *O*-antigens, lacking the short *O*-antigen chain (25–75 kDa). Acid hydrolysis of LPS samples removed the core and *O*-antigen from lipid A, revealing significantly lower amounts of polysaccharides in LPS from phage-resistant strains compared to the wild-type. Importantly, the complementation of *galE* restored polysaccharide levels to those of the wild-type (Fig. 4B).

### 3.5. *Phage-induced antibiotic susceptibility reprogramming in strains with deficient* galE

**Fig. 5.**
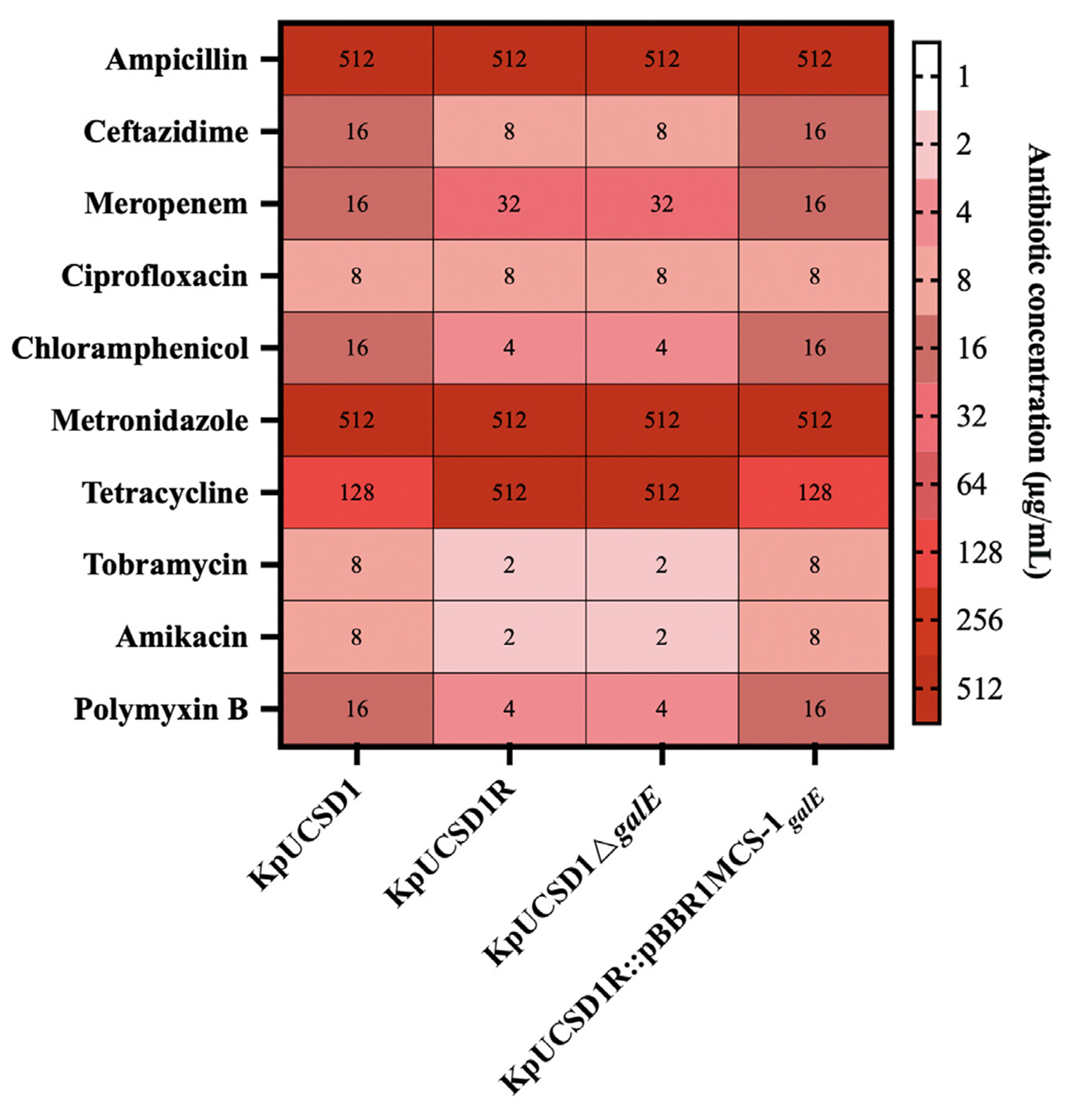
Antibiotic susceptibility profiles of KpUCSD1 and its derivatives.

We investigated the antimicrobial susceptibility of the phage-susceptible and -resistant *K. pneumoniae* strains. Both KpUCSD1R and KpUCSD1Δ*galE* exhibited significantly increased susceptibility to several classes of antibiotics compared to the wild-type KpUCSD1 and KpUCSD1R::pBBR1MCS-1*_galE_*, both of which carried functional *galE*. For instance, the MICs of polymyxin B and chloramphenicol in KpUCSD1 and KpUCSD1R::pBBR1MCS-1*_galE_* were 16 μg/mL, and a decrease to 4 μg/mL was observed in KpUCSD1R and KpUCSD1Δ*galE*. Similarly, the MICs of amikacin and to-bramycin were 8 μg/mL in KpUCSD1 and KpUCSD1R::pBBR1MCS-1*_galE_*, but decreased to 2 μg/mL in KpUCSD1R and KpUCSD1Δ*galE* (Fig. 5).

In contrast, *galE*-mediated phage resistance was also associated with reduced susceptibility to specific antibiotic classes, with KpUCSD1R and KpUCSD1Δ*galE* exhibiting increased MICs to meropenem and tetracycline relative to the *galE*-complemented strains (Fig. 5). Together, these findings demonstrate that *galE*-mediated phage resistance is associated with bidirectional antibiotic effects, depending on the antibiotic class.

## 4. Discussion

Understanding bacterial defence mechanisms is crucial for optimising phage therapy. Our study provides novel insights into phage resistance in *K. pneumoniae*, demonstrating the rapid emergence of resistance to the jumbo phage ΦKpUCSD1 (Fig. 1D). With a 348,922-bp genome, an icosahedral capsid, and a contractile tail, ΦKpUCSD1 has features typical of jumbo phages that target complex surface structures like LPS and capsular polysaccharides (Fig. 1A, B) [18,19]. Its genome encodes multiple receptor-binding proteins with predicted depolymerase activity, allowing recognition and degradation of diverse capsular types and supporting a broad host range [20,21]. Phage ΦKpUCSD1 was selected for characterisation due to its alignment with emerging *Klebsiella* jumbo phages, which display unusually broad host ranges and complex surface-recognition strategies. Such phages, including RaK2 [22], and K64-1 [23], are notable for their large genomes (≥300 kb), expanded structural gene cassettes, and ability to target multiple capsular and LPS serotypes via diverse receptor-binding proteins and depolymerases.

Our study demonstrates, for the first time, that *galE* in *K. pneumoniae* is essential for phage infection. Disruption of *galE* impairs phage adsorption and internalisation, likely due to defects in LPS biosynthesis, which is composed of lipid A, core oligosaccharide, and *O*-antigen (Fig. 3). The *galE* gene encodes for UDP-glucose 4-epimerase, which is involved in the conversion of UDP-galactose to UDP-glucose and *vice versa* [24]. UDP-galactose has been reported to serve as a donor for synthesising the core oligosaccharide and *O*-antigen of LPS [25]. The absence of *galE* results in truncated *O*-antigen and reduces phage binding, which is consistent with prior findings in *Salmonella typhimurium* [26,27], *Agrobacterium* sp. H13-3 [28], and *K. pneumoniae* [29]. While previous studies have associated capsule loss with phage resistance via mutations in *wzc, galU, wbaP*, and *wcaI* [30,31], our findings highlight the complexity and diversity of LPS-mediated phage resistance in *K. pneumoniae* (Fig. 4). Another recent work involving *Myoviridae* phage hvKpP3 reported that spontaneous hvKpP3-resistant mutants carried loss-of-function mutations in *wcaJ* or a glycosyltransferase gene, resulting in defective capsule or high-molecular-weight LPS and resistance to phage infection. Our study reveals a different mechanism, in which ΔKpUCSD1 resistance is driven by inactivation of *galE* that resulted in truncation of *O*-antigen, abolishing phage adsorption despite an otherwise intact capsule.

KpUCSD1 harbours an uncharacterised capsular type. Comparative analyses revealed mutations in the genes encoding for *galE* and *ubiH* among isolated phage-resistant mutants. Our data demonstrate that truncation of the *O*-antigen shifted the antibiotic susceptibility in *K. pneumoniae*, with resistant mutants (KpUCSD1R and KpUCSD1Δ*galE*) showing increased susceptibility to aminoglycosides, chloramphenicol and polymyxin B (Fig. 5). Previous studies have revealed a deep-rough LPS architecture in *K. pneumoniae* in which the loss of the short *O*-antigen exposes inner-core heptoses and lipid A phosphates, weakening Mg^2+^ /Ca^2+^ bridging and disrupting lateral LPS packing [32,33]. Consequently, polycationic aminoglycosides could enter the bacterial cell more readily through self-a uptake mechanism, hydrophobic molecules such as chloramphenicol could diffuse more efficiently across a disordered membrane, and polymyxin could gain enhanced access to lipid A [33–42]. Similar collateral sensitivities have been observed in *K. pneumoniae* mutants with defects in capsule or LPS biosynthesis, highlighting that many phage-resistance mutations impose envelope-associated fitness costs, as demonstrated by increased susceptibility to aminoglycosides and sulphonamides in previous studies [43].

It should be noted that, in contrast to most reports in the current literature describing increased antibiotic susceptibility following the evolution of phage resistance, we discovered here that phage resistance can also be associated with collateral reduction in antibiotic susceptibility. While *galE*-mediated LPS truncation imposed a clear fitness trade-off manifested as increased susceptibility to aminoglycosides, chloramphenicol, and polymyxin, it concurrently resulted in reduced susceptibility to meropenem and tetracycline. Consistent with this complexity, our recent work demonstrated that distinct phage resistance-conferring mutations in *K. pneumoniae* can produce opposing collateral effects, with insertion mutations in *rpoN* increasing polymyxin susceptibility, while mutations in *mutS* and *mutL* conferred polymyxin resistance [15]. Together, these findings underscore the complex and multifaceted nature of phage-antibiotic interactions in *K. pneumoniae*, highlighting that phage resistance can simultaneously generate therapeutic vulnerabilities and liabilities. This complexity cautions against assuming uniform antibiotic resensitisation following phage resistance, and importantly, underscores the need for precision phage– antibiotic combination therapy informed by mechanistic phage-antibiotic pairing strategies.

## 5. Conclusion

While phage therapy is increasingly recognised as a promising antimicrobial strategy to combat MDR bacterial infections, the emergence of phage resistance presents a significant clinical challenge. This study demonstrates, for the first time, that mutations in *galE* of *K. pneumoniae* lead to phage resistance by inhibiting phage adsorption. Importantly, a shift in antimicrobial susceptibility profile was observed in the phage-resistant mutants, including both increased and decreased susceptibility to different types of antibiotics. A comprehensive understanding of phage resistance mechanisms and associated antibiotic trade-offs will be crucial in guiding the optimal use of phage therapy in patients.

## Supplementary Material

1

Supplementary material associated with this article can be found, in the online version, at doi:10.1016/j.ijantimicag.2026.107738.

## Figures and Tables

**Table 1:** Mutations identified in phage-resistant KpUCSD1R.

Gene	Type of mutation	Effect of mutation	Ref/Alt	Nucleotide position	Effect	Gene product
*ubiH*	Single nucleotide polymorphism	Missense	T/G	626/1179	Glutamic acid (Glu) to alanine (Ala) at position 185	2-octaprenyl-6-methoxyphenol hydroxylase
*galE*	Insertion	Frameshift	T/TC	447/1017	Additional base shifted the reading frame of glutamic acid (Glu) at position 191	UDP-glucose 4-epimerase

## Data Availability

The data supporting the findings of this study are available within the article. The genomic data generated in this study have been deposited in the NCBI BioSample database under the following accessions: *Klebsiella pneumoniae* strain KpUCSD1 (BioSample accession SAMN33604660) and *K. pneumoniae* strain KpUCSD1R (BioSample accession SAMN53057282). Associated sequencing data are available through the corresponding NCBI entries. Any additional data can be obtained from the authors upon reasonable request.
