# Supplementary material for: Phage resistance bidirectionally altered antibiotic susceptibility in Klebsiella pneumoniae via galE mutation
Source: Int J Antimicrob Agents. Author manuscript; Available in PMC 2026 Jul 30. (PMC13420019; doi:10.1016/j.ijantimicag.2026.107738)
Supplement: 1 [file NIHMS2190697-supplement-1.docx]

**
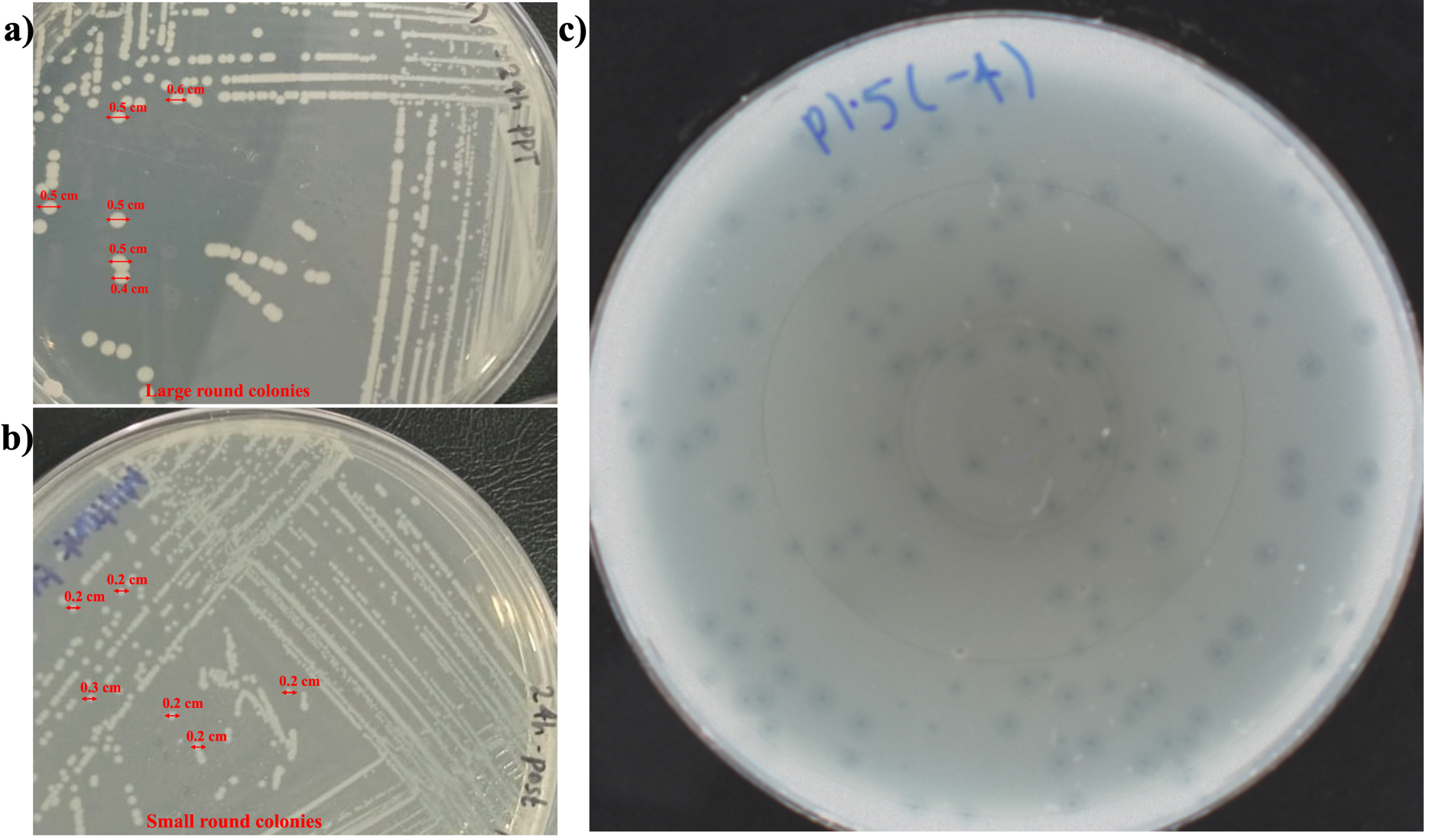
**

**Figure S1:** Two distinct bacterial colony morphologies, (a) large round colonies (~0.5 cm) and (b) small round colonies (~0.2 cm) were observed 24 h after treating KpUCSD1 with ΦKpUCSD1. (c) Plaque morphology of ΦKpUCSD1 on KpUCSD1 bacterial lawn showing clear and round plaques.

**
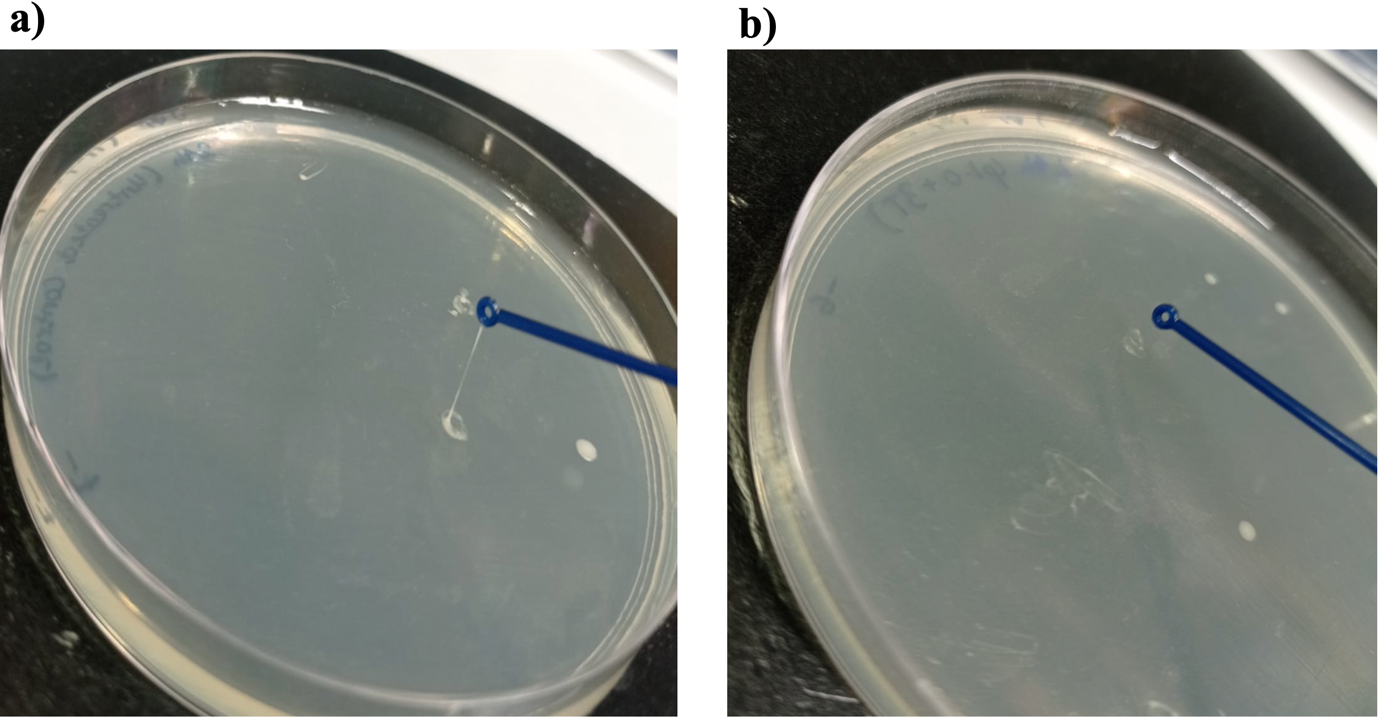
**

**Figure S2:** String test of the wild-type and phage-resistant mutant. (a) positive string test indicating mucoid and smooth phenotype of wild-type (KpUCSD1) colony. (b) The absence of string formation in dry and rough bacterial colonies observed with phage-resistant (KpUCSD1R) colony.

**
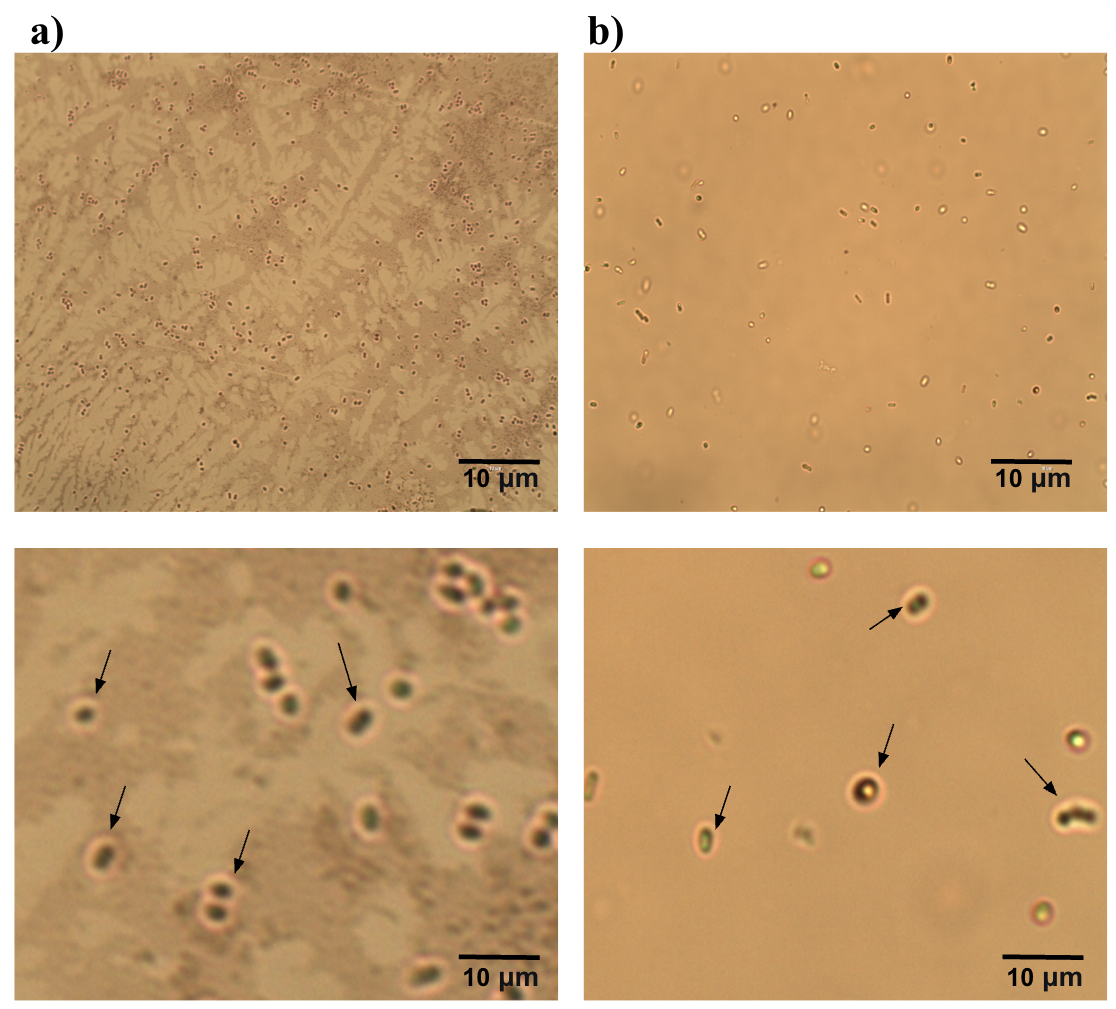
**

**Figure S3**: Maneval’s capsule staining of (**a)** KpUCSD1 (wild type) and (**b)** phage-resistant KpUCSD1R*.* The pink capsule was evident in both the wild-type and phage-resistant cells.

**
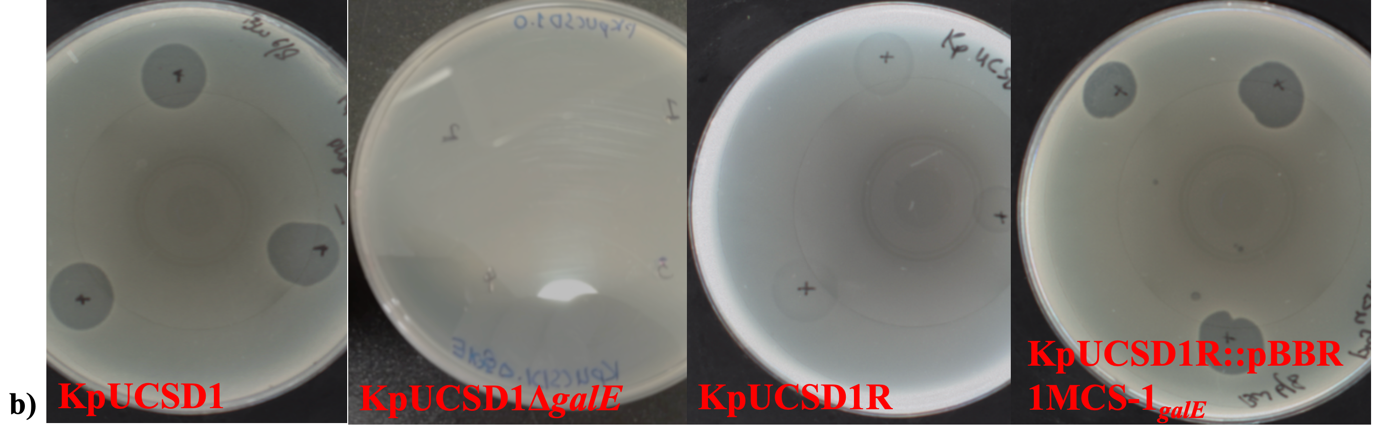
**

**Figure S4:** Direct-spot test of ΦKpUCSD1 on KpUCSD1 and its derivatives, with (a) the presence and absence of lysis zone, and (b) the visual representation of the assay with KpUCSD1, KpUCSD1Δ*galE*, KpUCSD1R, and KpUCSD1::pBBR1MCS-1*galE*.

**Figure 5**

**# Aligned_sequences: 2 (ubiH gene)**

**# 1: KpUCSD1**

**# 2: KpUCSD1R**

**# Matrix: EDNAFULL**

**# Gap_penalty: 10.0**

**# Extend_penalty: 0.5**

**#**

**# Length: 1179**

**# Identity: 1178/1179 (99.9%)**

**# Similarity: 1178/1179 (99.9%)**

**# Gaps: 0/1179 ( 0.0%)**

**# Score: 5886.0**

**#**

**#**

**#=======================================**

**KpUCSD1 1 TTAACGGGGAACCCAACCGAGGGTACGCTGCGCCAGCGCATCGCGCGCCG 50**

**||||||||||||||||||||||||||||||||||||||||||||||||||**

**KpUCSD1R 1 TTAACGGGGAACCCAACCGAGGGTACGCTGCGCCAGCGCATCGCGCGCCG 50**

**KpUCSD1 51 GGGTAAATAATTCCATCGCCATCAGACCGACGTTGCGCCCCGCCACCAGC 100**

**||||||||||||||||||||||||||||||||||||||||||||||||||**

**KpUCSD1R 51 GGGTAAATAATTCCATCGCCATCAGACCGACGTTGCGCCCCGCCACCAGC 100**

**KpUCSD1 101 GGCGCCCAACGGTTGGCAAACAGATGCACCAGCCCATCGGTCACGCCGAT 150**

**||||||||||||||||||||||||||||||||||||||||||||||||||**

**KpUCSD1R 101 GGCGCCCAACGGTTGGCAAACAGATGCACCAGCCCATCGGTCACGCCGAT 150**

**KpUCSD1 151 GGTGGCGGCTTTATCCCCTGCCCTGCGCGCCTGATAGCGGCACAGCAGCG 200**

**||||||||||||||||||||||||||||||||||||||||||||||||||**

**KpUCSD1R 151 GGTGGCGGCTTTATCCCCTGCCCTGCGCGCCTGATAGCGGCACAGCAGCG 200**

**KpUCSD1 201 GATAATGACCGACATCTTCGCCGCTGAGATGGGCGTCAGCGAGTAATTCC 250**

**||||||||||||||||||||||||||||||||||||||||||||||||||**

**KpUCSD1R 201 GATAATGACCGACATCTTCGCCGCTGAGATGGGCGTCAGCGAGTAATTCC 250**

**KpUCSD1 251 GCCAGGCTCATCACATCGCGCAGGCCGAGATTAAAGCCCTGCCCGGCGAT 300**

**||||||||||||||||||||||||||||||||||||||||||||||||||**

**KpUCSD1R 251 GCCAGGCTCATCACATCGCGCAGGCCGAGATTAAAGCCCTGCCCGGCGAT 300**

**KpUCSD1 301 CGGGTGCAGCGTCTGCGCCGCGTTGCCGACCAGCGCCAGGCGGTGCGACA 350**

**||||||||||||||||||||||||||||||||||||||||||||||||||**

**KpUCSD1R 301 CGGGTGCAGCGTCTGCGCCGCGTTGCCGACCAGCGCCAGGCGGTGCGACA 350**

**KpUCSD1 351 CCGCGCGGCTGGCGGTGGTGAGGGCCAGCGGATAGACGCTGCGCTTCCCG 400**

**||||||||||||||||||||||||||||||||||||||||||||||||||**

**KpUCSD1R 351 CCGCGCGGCTGGCGGTGGTGAGGGCCAGCGGATAGACGCTGCGCTTCCCG 400**

**KpUCSD1 401 GCGTGGGTAATACGCCCCAGCCGCCAGCCGAACGCCTGCTGCAGCTCCTG 450**

**||||||||||||||||||||||||||||||||||||||||||||||||||**

**KpUCSD1R 401 GCGTGGGTAATACGCCCCAGCCGCCAGCCGAACGCCTGCTGCAGCTCCTG 450**

**KpUCSD1 451 ACAAAAGCGTTCGTCAGACCAGCTCTGCACCTCATCGCGGCGCGACTGCG 500**

**||||||||||||||||||||||||||||||||||||||||||||||||||**

**KpUCSD1R 451 ACAAAAGCGTTCGTCAGACCAGCTCTGCACCTCATCGCGGCGCGACTGCG 500**

**KpUCSD1 501 GATGACACCACACCAGCGAGCAGCGCCCTTGCGACATCGGCAGCATCGCC 550**

**||||||||||||||||||||||||||||||||||||||||||||||||||**

**KpUCSD1R 501 GATGACACCACACCAGCGAGCAGCGCCCTTGCGACATCGGCAGCATCGCC 550**

**KpUCSD1 551 AGCGGGCCATGCTCGGTAAAGCGCTCGAAGGCGCGACCTTCGTGGGGCAA 600**

**||||||||||||||||||||||||||||||||||||||||||||||||||**

**KpUCSD1R 551 AGCGGGCCATGCTCGGTAAAGCGCTCGAAGGCGCGACCTTCGTGGGGCAA 600**

**KpUCSD1 601 CGCGGTGCTGACGTTAGCAATGATCTCTATCTGCTCATACGGCTGCTGCT 650**

**|||||||||||||||||||||||||.||||||||||||||||||||||||**

**KpUCSD1R 601 CGCGGTGCTGACGTTAGCAATGATCGCTATCTGCTCATACGGCTGCTGCT 650**

**KpUCSD1 651 GCCAGCTGATGCCGCAGCGGGCGCCCAGTGCCGAACGGGAACCGTCCGCC 700**

**||||||||||||||||||||||||||||||||||||||||||||||||||**

**KpUCSD1R 651 GCCAGCTGATGCCGCAGCGGGCGCCCAGTGCCGAACGGGAACCGTCCGCC 700**

**KpUCSD1 701 GCCACCAGCAGCTTACCGTTAATGATTTCGCCGCCCTCAAGCGTAAGGCT 750**

**||||||||||||||||||||||||||||||||||||||||||||||||||**

**KpUCSD1R 701 GCCACCAGCAGCTTACCGTTAATGATTTCGCCGCCCTCAAGCGTAAGGCT 750**

**KpUCSD1 751 GACGCTCTCCTGGCTGCGACTGACCGCTTCCACTTTCGCCGGACAGTGCA 800**

**||||||||||||||||||||||||||||||||||||||||||||||||||**

**KpUCSD1R 751 GACGCTCTCCTGGCTGCGACTGACCGCTTCCACTTTCGCCGGACAGTGCA 800**

**KpUCSD1 801 GCGTGACGCCCGGCGCCTCGCGCAGCAGACCAAACAGCCGCTGGCCGACA 850**

**||||||||||||||||||||||||||||||||||||||||||||||||||**

**KpUCSD1R 801 GCGTGACGCCCGGCGCCTCGCGCAGCAGACCAAACAGCCGCTGGCCGACA 850**

**KpUCSD1 851 TCGTGGAGTTCAACCACCTGGCCCAGCGCCGACAGGCCATAATCCGCCGC 900**

**||||||||||||||||||||||||||||||||||||||||||||||||||**

**KpUCSD1R 851 TCGTGGAGTTCAACCACCTGGCCCAGCGCCGACAGGCCATAATCCGCCGC 900**

**KpUCSD1 901 CGCCAGATTCACAAACCCGGCATGACCGCGGTCGCTGACGTGTACTCGCT 950**

**||||||||||||||||||||||||||||||||||||||||||||||||||**

**KpUCSD1R 901 CGCCAGATTCACAAACCCGGCATGACCGCGGTCGCTGACGTGTACTCGCT 950**

**KpUCSD1 951 GGATGGGCGTCGCGCGTTCGGCGAGGCGTTGCCAGATACCGATACGCGCC 1000**

**||||||||||||||||||||||||||||||||||||||||||||||||||**

**KpUCSD1R 951 GGATGGGCGTCGCGCGTTCGGCGAGGCGTTGCCAGATACCGATACGCGCC 1000**

**KpUCSD1 1001 AGCTGCTGGCAGGTGCCCGCAGCCAGGGCGATCGCCCGGTCGTCAAAGCC 1050**

**||||||||||||||||||||||||||||||||||||||||||||||||||**

**KpUCSD1R 1001 AGCTGCTGGCAGGTGCCCGCAGCCAGGGCGATCGCCCGGTCGTCAAAGCC 1050**

**KpUCSD1 1051 AGGGTGGCGTGACGAATGCGGATCCTGCGCTTCAATGAGGTGGACCGGCA 1100**

**||||||||||||||||||||||||||||||||||||||||||||||||||**

**KpUCSD1R 1051 AGGGTGGCGTGACGAATGCGGATCCTGCGCTTCAATGAGGTGGACCGGCA 1100**

**KpUCSD1 1101 GCGCGCCGCCGGTCAAACGGGAGATGGCCAGCGCGAGCGTCGCCCCGGTC 1150**

**||||||||||||||||||||||||||||||||||||||||||||||||||**

**KpUCSD1R 1101 GCGCGCCGCCGGTCAAACGGGAGATGGCCAGCGCGAGCGTCGCCCCGGTC 1150**

**KpUCSD1 1151 ATCCCGCCCCCGACGATCAGCACGCTCAT 1179**

**|||||||||||||||||||||||||||||**

**KpUCSD1R 1151 ATCCCGCCCCCGACGATCAGCACGCTCAT 1179**

**>KpUCSD1**

**2-octaprenyl-6-methoxyphenol hydroxylase ubiH**

**TTAACGGGGAACCCAACCGAGGGTACGCTGCGCCAGCGCATCGCGCGCCGGGGTAAATAATTCCATCGCCATCAGACCGACGTTGCGCCCCGCCACCAGCGGCGCCCAACGGTTGGCAAACAGATGCACCAGCCCATCGGTCACGCCGATGGTGGCGGCTTTATCCCCTGCCCTGCGCGCCTGATAGCGGCACAGCAGCGGATAATGACCGACATCTTCGCCGCTGAGATGGGCGTCAGCGAGTAATTCCGCCAGGCTCATCACATCGCGCAGGCCGAGATTAAAGCCCTGCCCGGCGATCGGGTGCAGCGTCTGCGCCGCGTTGCCGACCAGCGCCAGGCGGTGCGACACCGCGCGGCTGGCGGTGGTGAGGGCCAGCGGATAGACGCTGCGCTTCCCGGCGTGGGTAATACGCCCCAGCCGCCAGCCGAACGCCTGCTGCAGCTCCTGACAAAAGCGTTCGTCAGACCAGCTCTGCACCTCATCGCGGCGCGACTGCGGATGACACCACACCAGCGAGCAGCGCCCTTGCGACATCGGCAGCATCGCCAGCGGGCCATGCTCGGTAAAGCGCTCGAAGGCGCGACCTTCGTGGGGCAACGCGGTGCTGACGTTAGCAATGATCTCTATCTGCTCATACGGCTGCTGCTGCCAGCTGATGCCGCAGCGGGCGCCCAGTGCCGAACGGGAACCGTCCGCCGCCACCAGCAGCTTACCGTTAATGATTTCGCCGCCCTCAAGCGTAAGGCTGACGCTCTCCTGGCTGCGACTGACCGCTTCCACTTTCGCCGGACAGTGCAGCGTGACGCCCGGCGCCTCGCGCAGCAGACCAAACAGCCGCTGGCCGACATCGTGGAGTTCAACCACCTGGCCCAGCGCCGACAGGCCATAATCCGCCGCCGCCAGATTCACAAACCCGGCATGACCGCGGTCGCTGACGTGTACTCGCTGGATGGGCGTCGCGCGTTCGGCGAGGCGTTGCCAGATACCGATACGCGCCAGCTGCTGGCAGGTGCCCGCAGCCAGGGCGATCGCCCGGTCGTCAAAGCCAGGGTGGCGTGACGAATGCGGATCCTGCGCTTCAATGAGGTGGACCGGCAGCGCGCCGCCGGTCAAACGGGAGATGGCCAGCGCGAGCGTCGCCCCGGTCATCCCGCCCCCGACGATCAGCACGCTCAT**

**>KpUCSD1R**

**2-octaprenyl-6-methoxyphenol hydroxylase ubiH**

**TTAACGGGGAACCCAACCGAGGGTACGCTGCGCCAGCGCATCGCGCGCCGGGGTAAATAATTCCATCGCCATCAGACCGACGTTGCGCCCCGCCACCAGCGGCGCCCAACGGTTGGCAAACAGATGCACCAGCCCATCGGTCACGCCGATGGTGGCGGCTTTATCCCCTGCCCTGCGCGCCTGATAGCGGCACAGCAGCGGATAATGACCGACATCTTCGCCGCTGAGATGGGCGTCAGCGAGTAATTCCGCCAGGCTCATCACATCGCGCAGGCCGAGATTAAAGCCCTGCCCGGCGATCGGGTGCAGCGTCTGCGCCGCGTTGCCGACCAGCGCCAGGCGGTGCGACACCGCGCGGCTGGCGGTGGTGAGGGCCAGCGGATAGACGCTGCGCTTCCCGGCGTGGGTAATACGCCCCAGCCGCCAGCCGAACGCCTGCTGCAGCTCCTGACAAAAGCGTTCGTCAGACCAGCTCTGCACCTCATCGCGGCGCGACTGCGGATGACACCACACCAGCGAGCAGCGCCCTTGCGACATCGGCAGCATCGCCAGCGGGCCATGCTCGGTAAAGCGCTCGAAGGCGCGACCTTCGTGGGGCAACGCGGTGCTGACGTTAGCAATGATCGCTATCTGCTCATACGGCTGCTGCTGCCAGCTGATGCCGCAGCGGGCGCCCAGTGCCGAACGGGAACCGTCCGCCGCCACCAGCAGCTTACCGTTAATGATTTCGCCGCCCTCAAGCGTAAGGCTGACGCTCTCCTGGCTGCGACTGACCGCTTCCACTTTCGCCGGACAGTGCAGCGTGACGCCCGGCGCCTCGCGCAGCAGACCAAACAGCCGCTGGCCGACATCGTGGAGTTCAACCACCTGGCCCAGCGCCGACAGGCCATAATCCGCCGCCGCCAGATTCACAAACCCGGCATGACCGCGGTCGCTGACGTGTACTCGCTGGATGGGCGTCGCGCGTTCGGCGAGGCGTTGCCAGATACCGATACGCGCCAGCTGCTGGCAGGTGCCCGCAGCCAGGGCGATCGCCCGGTCGTCAAAGCCAGGGTGGCGTGACGAATGCGGATCCTGCGCTTCAATGAGGTGGACCGGCAGCGCGCCGCCGGTCAAACGGGAGATGGCCAGCGCGAGCGTCGCCCCGGTCATCCCGCCCCCGACGATCAGCACGCTCAT**

**Table S1:** Primers used for gene complementation and knockout.

| Primer | Sequence (5’ – 3’) |
| --- | --- |
| *ubiH*_SacI_FW primer | ACTGA**GAGCTC**CATTCCACCGCCAACAATG |
| *ubiH*_ApaI_RV primer | ATCGT**GGGCCC**GAAGAAGGCGGATGAGATCG |
| *galE*_SacI_FW primer | ACTGA**GAGCTC**CGATGTGGATGGTCAACG |
| *galE*_ApaI_RV primer | ATCGT**GGGCCC**GTTACACTTATCAGCTCGGC |
| ApaI_IsceI_*galE*_DXO_Up_Fw | ATCGT**GGGCCC**TAGGGATAACAGGGTAAT*GCGCCATTTCGTCCAGGTTG* |
| *galE*_DXOUpRv | CGAAGCAGCTCCAGCCAACA*CCCGACCGAAGATGGTACC* |
| *galE*_DXODown_Fw | CTAAGGAGGATATTCATATGGACC*GCATGGCAGAGACTAATTTCAG* |
| SpeI_IsceI_*galE*_DXO_DownRv | ACTGA**ACTAGT**TAGGGATAACAGGGTAAT*GCCCTATGTCACACTTTTCAC* |
| *galE*_Cnf_KO_Fw | GTTCCTTAGTCTGGATAACCC |
| *galE*_Cnf_KO_Rv | GCGCCAAACAAACCTAAGTC |
